# Function-Altering SNPs in the Human Multidrug Transporter Gene *ABCB1* Identified Using a *Saccharomyces*-Based Assay

**DOI:** 10.1371/journal.pgen.0030039

**Published:** 2007-03-09

**Authors:** Hotcherl Jeong, Ira Herskowitz, Deanna L Kroetz, Jasper Rine

**Affiliations:** 1 Department of Molecular and Cell Biology, University of California Berkeley, Berkeley, California, United States of America; 2 Department of Biochemistry and Biophysics, University of California San Francisco, San Francisco, California, United States of America; 3 Institute for Human Genetics, University of California San Francisco, San Francisco, California, United States of America; Brandeis University, United States of America; 4 Department of Biopharmaceutical Sciences, University of California San Francisco, San Francisco, California, United States of America

## Abstract

The human *ABCB1* (*MDR1*)-encoded multidrug transporter P-glycoprotein (P-gp) plays a major role in disposition and efficacy of a broad range of drugs including anticancer agents. *ABCB1* polymorphisms could therefore determine interindividual variability in resistance to these drugs. To test this hypothesis we developed a *Saccharomyces*-based assay for evaluating the functional significance of *ABCB1* polymorphisms. The P-gp reference and nine variants carrying amino-acid–altering single nucleotide polymorphisms (SNPs) were tested on medium containing daunorubicin, doxorubicin, valinomycin, or actinomycin D, revealing SNPs that increased (M89T, L662R, R669C, and S1141T) or decreased (W1108R) drug resistance. The R669C allele's highly elevated resistance was compromised when in combination with W1108R. Protein level or subcellular location of each variant did not account for the observed phenotypes. The relative resistance profile of the variants differed with drug substrates. This study established a robust new methodology for identification of function-altering polymorphisms in human multidrug transporter genes, identified polymorphisms affecting P-gp function, and provided a step toward genotype-determined dosing of chemotherapeutics.

## Author Summary

Patients often show varied drug responses ranging from lack of therapeutic efficacy to life-threatening adverse drug reactions. Drug therapy would be greatly improved if it were possible to predict individual drug sensitivity and tailor drugs to patients' genetic makeup. Like all other organisms, humans have a set of transporters and enzymes to detoxify and eliminate foreign molecules including drugs. Understanding the function of genetic variants in these proteins is a key goal toward personalized medicine. To that end, we examined the functional consequences of naturally occurring genetic variants in P-glycoprotein, the most versatile human multidrug transporter. A novel method was developed and employed that can identify function-altering variants in human transporters. This methodology was robust and powerful in that the functional effect of genetic variants can be directly assessed in yeast where all confounding variables in humans are excluded. Surprisingly, the majority of single amino acid substitutions were found to cause alterations in resistance to three tested anticancer agents. This study extends the impact of yeast-based medical research to a new niche, pharmacogenomics.

## Introduction

Patients vary widely in their drug responses including unpredicted adverse drug reactions that cause a significant loss of lives and a huge toll on health-care costs [1]. Rational selection and dosage optimization of anticancer agents are particularly important due to their narrow therapeutic index and inherent cytotoxicity. Membrane transporters affect drug disposition and response by determining whether or not the level of drug is maintained within the therapeutic index. Of the known human transporters, P-glycoprotein (P-gp) is of particular clinical relevance in that this multidrug efflux pump has a broad range of substrates, including structurally and functionally divergent drugs in common clinical use [2–4]. P-Gp belongs to the ATP-binding cassette (ABC) superfamily [5] and is encoded by the human *ABCB1* gene (also known as multidrug resistance 1 gene [MDR1]). Multidrug resistance caused by *ABCB1* amplification is a major obstacle in cancer chemotherapy. In fact, the *ABCB1* gene was originally identified because of its amplification in tumor cells that had acquired cross-resistance to multiple cytotoxic anticancer agents [2,6–9]. P-Gp is expressed in many tissues, suggestive of a broad physiological role [10,11] and functions by pumping cytotoxic drugs and xenotoxins out of cells into the intestinal lumen, bile, and urine, and thus limiting distribution of such compounds to other organs.

Genetic heterogeneity of the *ABCB1* gene may be a potent determinant of interindividual variability in resistance to multiple drugs including anticancer agents. Furthermore, P-gp can act alone or in combination with other genetic variants, particularly polymorphisms in CYP3A4, a cytochrome P450 monooxygenase that metabolizes a wide range of drugs [12,13]. Naturally occurring null mutations in P-gp have been reported in mice and dogs but not in humans [14,15]. Animals carrying a null *ABCB1* variant are viable unless challenged by drugs that are substrates for P-gp. Likewise, there may be unidentified human *ABCB1* variants that cause a total loss of function. Numerous *ABCB1* single nucleotide polymorphisms (SNPs) have been identified. However, the correlation of SNPs with *ABCB1* expression and P-gp function in clinical pharmacokinetics has been inconclusive. A synonymous 3435C>T SNP has been heavily studied, but its function remains under debate [16]. Moreover, to date there have been no naturally occurring nonsynonymous substitutions with a validated functional consequence [17]. Robust functional assays of P-gp variants at the cellular and molecular levels are needed to address their impact on clinical pharmacokinetics.

Since human populations are outbred, and each individual is heterozygous for several million polymorphisms, the impact of *ABCB1* variants is difficult to separate from the potential contributions of other variations in an individual. Yeast cells offer an excellent context for functional analysis of foreign eukaryotic transport proteins [18]. Expressing human proteins and their variants in yeast allows the function of individual variants to be assessed directly. The human P-gp can be functionally expressed in the yeast Saccharomyces cerevisiae, where it exports at least some of the same compounds that it exports in human cells [19]. A typical assay for human P-gp function in yeast involves testing its ability to restore growth to cells in the presence of compounds that would otherwise block their growth. This functional complementation in yeast allows the impact of *ABCB1* variants found in human populations to be assessed.

This study tested the functional consequences of *ABCB1* genetic variants found in ethnically diverse populations (Figure S1) [20]. From this dataset (http://pharmacogenetics.ucsf.edu or http://www.pharmgkb.org), we prioritized nonsynonymous SNPs by their predicted impact on P-gp function, selected ten haplotypes carrying high-priority SNP(s), and determined the level of resistance caused by these *ABCB1* variants to clinically important drugs. For those variants that altered function, subsequent experiments tested the mechanism of these effects.

## Results

### The Human *ABCB1* Gene Functioned in Hyper-Sensitized Yeast Cells

**Figure 1:**
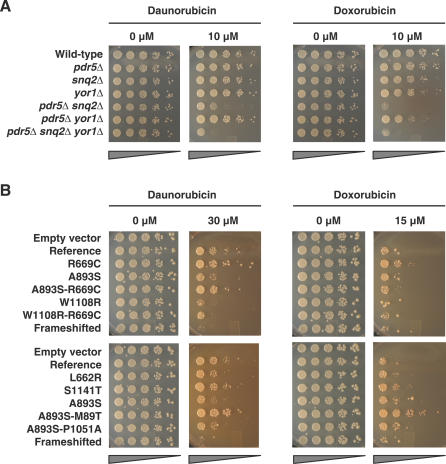
Functional Complementation of Human cDNAs for P-Gp Variants in a Drug-Sensitive Yeast Strain (A) Differences in drug sensitivity of yeast strains. Yeast strains tested included mutants in which combinations of three major endogenous multidrug transporter genes, *PDR5*, *SNQ2*, and *YOR1*, were deleted. Cultures from each strain were adjusted to the same cell concentration and serially diluted 5-fold. The genotypes of the strains tested are shown in Table S1. The *pdr5 snq2 yor1* triple mutant was consistently the most sensitive strain to daunorubicin and doxorubicin. (B) Restoration of drug resistance in the hyper-sensitized yeast strain by P-gp variants. Cultures from each strain were adjusted to the same cell concentration and serially diluted 5-fold. Variants restored drug resistance of the hyper-sensitized yeast strain to varying degrees. The strains tested are *pdr5 snq2 yor1* with plasmids carrying the indicated variants (Table S1). A frame-shifted P-gp was used as a negative control.

As the first step toward functional analysis of the nonsynonymous variants of human P-gp, we tested the sensitivity of yeast strains harboring mutations in major endogenous multidrug transporter genes, *PDR5*, *SNQ2*, and *YOR1*. Combinatorial deletions of these three genes confer sensitivity to a variety of toxic compounds including two anticancer agents, daunorubicin and doxorubicin, which are substrates for human P-gp [21]. The double mutant *pdr5 yor1* (JRY8008) displayed increased sensitivity relative to wild-type cells toward doxorubicin, whereas another double mutant *pdr5 snq2* (JRY8004) displayed increased sensitivity toward daunorubicin and doxorubicin. The strain that exhibited the greatest drug sensitivity was the *pdr5 snq2 yor1* triple deletion mutant (JRY8012) (Figure 1A) (see Table S1 for the strain list). This result was reminiscent of bacterial multidrug efflux pumps that produce greater drug resistance in combination than alone [22].

To address the function of human P-gp in yeast, we used a plasmid (pJR2702) that contains a cDNA for the human *ABCB1* gene expressed from the promoter for the *S. cerevisiae STE6* gene on a multicopy vector [19]. The yeast *STE6* gene encodes an ABC transporter that mediates the export of the **a**-factor pheromone in *MAT* ***a*** cells. The cloned cDNA carried the G185V SNP of *ABCB1*, and therefore site-directed mutagenesis was used to restore it to the most common allele, referred to as the *ABCB1* reference allele in the Pharmacogenetics of Membrane Transporters dataset (pJR2703) (http://pharmacogenetics.ucsf.edu or http://www.pharmgkb.org). Cells expressing the *ABCB1* reference cDNA from the multicopy plasmid in the *pdr5 snq2 yor1* strain showed highly increased resistance towards daunorubicin and doxorubicin relative to that of the *pdr5 snq2 yor1* strain (Figure 1B). Thus the P-gp reference was functionally expressed in these yeast cells.

### Prediction of Functional Impact of SNPs by Evolutionary Conservation and Chemical Dissimilarity

The Pharmacogenetics of Membrane Transporters study identified fourteen nonsynonymous SNPs in 247 healthy individuals from an ethnically diverse population (Figure S1) [20]. These SNPs comprised 25 haplotypes including 15 haplotypes in which the phase relationship of the SNPs was inferred but not directly resolved. SNPs were prioritized for functional analysis by two criteria: the degree of evolutionary conservation [23] and the biochemical severity of the alteration. The extent of evolutionary sequence conservation and thus inferred constraint at a particular residue was observed across ten mammalian species. The severity of missense changes was estimated by the Grantham scale [24], which formulates the difference in codon substitutions based on chemical dissimilarity of the encoded amino acids. Grantham values range between 5 and 215, with higher values indicating more radical chemical changes.

Out of the 14 nonsynonymous SNPs in the dataset [20], we chose seven SNPs for functional characterization (Table 1). We first focused on the five SNPs with highest Grantham values (>80): M89T, L662R, R669C, A893S, and W1108R. The M89T polymorphic site was not evolutionarily conserved, but the other four sites were highly conserved. In addition, the P1051A SNP was chosen because of its conservation despite a low Grantham value, and the S1141T SNP was included due to its relatively high allele frequency (11% in African Americans) and evolutionary conservation. Although A893S, S1141T, and R669C SNPs are common variants (minor allele frequency ≥1% in at least one major ethnic group), the remaining four chosen variants are observed only once among 494 alleles from different populations. These rare variants (minor allele frequency <1%) were included because rare adverse drug reactions may be due to highly penetrant but rare variants. The alignment and allele count of *ABCB1* haplotypes based on the 14 nonsynonymous SNPs identified in the previous resequencing project are presented in Table S2. From the standpoint of functional impact, the R669C SNP was particularly interesting. First, this Arg-to-Cys substitution had the highest Grantham value (180) among the fourteen SNPs. Second, this SNP was observed twice in the African American population exhibiting a 1% allele frequency, whereas the four chosen rare variants occurred only once. Third, the R669C SNP may be in phase with the W1108R variant. One of the two R669C SNPs was detected in an individual whose *ABCB1* gene also contained the W1108R variant, potentially resulting in haplotype R669C-W1108R. This observation prompted us to test whether a R669C-W1108R allele had a unique phenotype relative to alleles carrying each individual SNP.

**Table 1:**
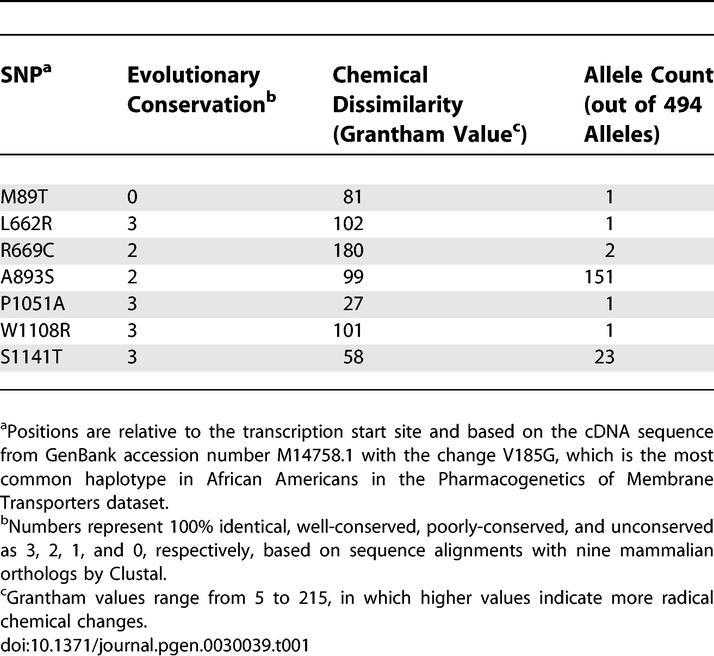
Features of the *ABCB1* Variants Analyzed in This Study

### P-Gp Variants Exhibited Altered Resistance to Two Anticancer Agents

**Figure 2:**
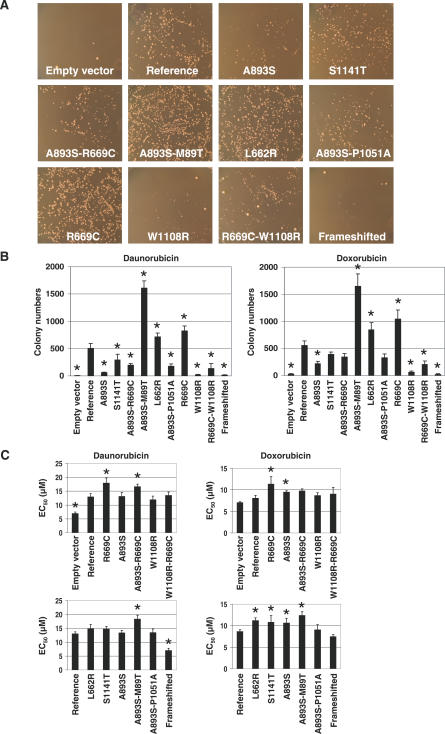
Alterations in Resistance of P-Gp Variants to Daunorubicin and Doxorubicin (A) Drug resistance of P-gp variants measured with a plating assay on solid medium. Cultures from each strain were adjusted to the same cell concentration and spread on plates carrying daunorubicin (27.5 μM) or doxorubicin (12.5 μM). Yeast expressing P-gp variants survived on these high-concentration drug plates to varying degrees. Daunorubicin plates for each of the P-gp variants are presented. The corresponding plates for doxorubicin showed similar variability. (B) Drug resistance of P-gp variants represented by colony numbers surviving on the high-concentration drug plates. Each value represents the average ± standard deviation of three measurements. The values with significant differences (*p* < 0.05) are marked with *. (C) Drug resistance of P-gp variants in liquid medium. Each EC_50_ value represents the average ± standard deviation of three or four measurements. The values with significant differences (*p* < 0.05) are marked with *.

We constructed plasmids expressing P-gp variants by site-directed mutagenesis on the reference plasmid to evaluate the effect of selected SNPs and their combinations on P-gp function. These plasmids (pJR2703–pJR2712), along with two control vectors (YEp352 and pJR2713), were transformed into the *pdr5 snq2 yor1* strain (JRY8012). These yeast strains carrying plasmids with *ABCB1* variants (JRY8025–JRY8036) were examined for their level of resistance to daunorubicin and doxorubicin on solid medium. Different P-gp variants displayed higher levels of resistance (A893S-M89T, L662R, and R669C) or lower levels of resistance (A893S, S1141T, A893S-R669C, A893S-P1051A, W1108R, and W1108R-R669C) relative to the P-gp reference (Figure 2A and 2B). The alleles varied widely in their ability to survive on high concentrations of daunorubicin and doxorubicin. The replacement of Arg669 by Cys led to one of the most drastic gain-of-function effects on the ability of P-gp to confer drug resistance. This allele's elevated resistance was compromised when in combination with W1108R. Cells expressing truncated P-gp (see Materials and Methods) were indistinguishable from cells transformed with an empty vector with respect to drug resistance.

To quantify the extent of drug cytotoxicity in liquid medium, median effective concentration (EC_50_) values were measured for daunorubicin and doxorubicin for each P-gp variant in liquid culture (Figure 2C). For the majority of the variants, these results were consistent with those observed in the plate assay. However, the plate assay was more sensitive, allowing variants that were indistinguishable from each other in the liquid assay to be ranked. There was a discrepancy between the two drug resistance phenotypes with the A893S and A893S-R669 variants: the variants showed a slightly higher level of drug resistance relative to that of the reference in the liquid assay, but a lower survival in the plate assay. This difference presumably reflects the nature of the two assays: the plate assay measures the level of cell survival on a relatively high fixed concentration of the drug, whereas the liquid assay determines growth rate over multiple drug concentrations. In the plate assay, all variants for daunorubicin and six variants for doxorubicin exhibited statistically significant differences (*p* < 0.05) (Figure 2B; Table S3). In the liquid assay, three variants for daunorubicin (A893S-R669C, A893S-M89T, and R669C) and five variants for doxorubicin (A893S, S1141T, A893S-M89T, L662R, and R669C) exhibited statistically significant increases in EC_50_ values (*p* < 0.05) (Figure 2C; Table S4).

### P-Gp and Its Variants Had Similar Protein Level and Subcellular Localization

**Figure 3:**
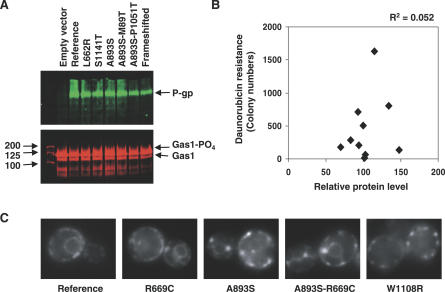
Protein Level and Subcellular Localization of P-Gp Variants (A) Immunoblot analysis for P-gp variants expressed in the plasma membrane fraction of yeast cells. Two-color immunoblot detection was used to quantify the level of P-gp variants compared with the level of yeast Gas1, a membrane protein-loading control. The positions of molecular standards (in kilodaltons) are indicated at the left. The arrows at the right indicate the position of the P-gp and Gas1 proteins expressed in yeast cells. All P-gp variants were expressed within a 1.5-fold range from the reference P-gp. The average ± standard deviation of three measurements of relative protein levels is 103 ± 19 for A893S, 83 ± 17 for S1141T, 94 ± 16 for A893S-R669C, 115 ± 20 for A893S-M89T, 93 ± 19 for L662R, 70 ± 22 for A893S-P1051A, 134 ± 25 for R669C, 102 ± 18 for W1108R, 148 ± 24 for R669C-W1108R, and 53 ± 13 for frame-shifted, relative to the average of reference P-gp set to 100. The cells expressing the frame-shifted P-gp produced the least amount. The truncated length of the frame-shifted protein was not recognized in this gel. (B) The relationship between daunorubicin resistance and P-Gp protein levels. The x-axis shows the relative protein level for each variant protein with the level for the reference set at 100. The y-axis shows daunorubicin resistance represented as colony numbers surviving on the high-concentration daunorubicin plates. (C) Subcellular localization of GFP-fused P-Gp variants. P-Gp variants were localized to the plasma membrane and the vacuolar membrane in yeast cells. The images were taken when the cells reached midexponential phase (OD_600_ = 0.7). The cells expressing each P-gp variant fluoresced to similar extents, regardless of the drug resistance phenotypes.

To determine whether the observed differences in drug resistance were due to differences in protein level, we measured the protein level of each P-gp variant by immunoblotting. The mouse anti-P-gp antibody detected P-gps with an apparent molecular mass of 125 kDa, the expected size of unglycosylated P-gp, in membranes from yeast cells transformed with plasmids carrying reference and variant *ABCB1* genes, but not in membranes from control cells transformed with an empty vector. The amount of P-gp reference and variants differed by no more than 1.5-fold (Figure 3A). The correlation coefficient of the extent of daunorubicin cytotoxicity of each variant relative to the protein level of each variant was 0.227 (Figure 3B). Thus the P-gp variants were present at comparable levels and altered drug cytotoxicity in the variants was not due to the differences in protein levels for P-gp.

In principle, the differing drug resistance of the variants might reflect differences in their subcellular localization if the SNPs affected the P-gp trafficking. To test this possibility, strains carrying green fluorescent protein (GFP) fused in frame to the C terminus of each P-gp variant were evaluated for their subcellular localization patterns. Fluorescence microscopy indicated that the fusion proteins were localized to both the plasma membrane and the vacuolar membrane in living cells (Figure 3C). The localization patterns were growth-phase–dependent: GFP fluorescence was observed mostly in the plasma membrane in mid-log phase cells and became more concentrated in vacuoles when the cells were grown into the stationary phase. The cells carrying each of the GFP-fused P-gp variants fluoresced to similar extents from the same subcellular location under each growth phase. Thus differences in subcellular localization were unlikely to underlie the differences in drug resistance associated with the variants.

### The Relative Resistance Profile of the Variants Differed with the Drug Substrates

**Figure 4:**
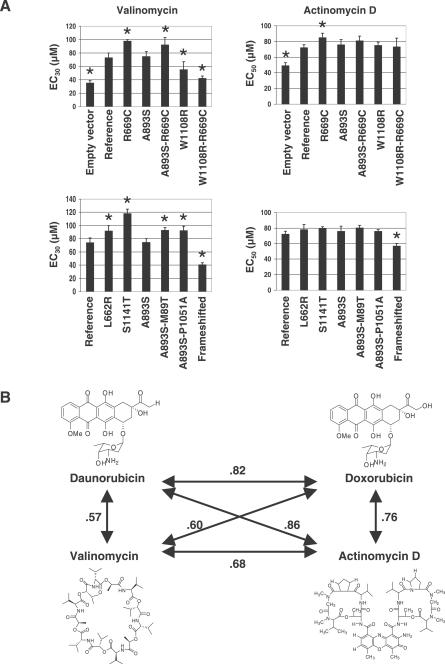
Comparison of Resistance of the P-Gp Variants to Different Drug Substrates (A) Alterations in resistance of P-gp variants to valinomycin and actinomycin D. The degree of resistance of the variants to valinomycin and actinomycin D are presented as EC_30_ and EC_50_ values, respectively. Each EC_30_ or EC_50_ value represents the average ± standard deviation of three measurements. The values with significant differences (*p* < 0.05) are marked with *. (B) Correlation of the drug resistance profiles for different drug substrates. The correlation coefficients for each pair of EC_50_ (or EC_30_) values are presented.

The relative resistance of each P-gp variant to the structurally similar drugs, daunorubicin and doxorubicin, were quite similar (Figure 2). Because P-gp can confer cellular resistance to a variety of cytotoxic drugs, we tested whether P-gp variants might exhibit different resistance profiles when tested with additional P-gp substrates, valinomycin and actinomycin D, which are structurally dissimilar from daunorubicin and doxorubicin. Due to the limited solubility of valinomycin in synthetic (CSM)–Ura culture medium, determining the EC_50_ values was not possible. However, determining the EC_30_ proved sufficient to distinguish among the P-gp variants for valinomycin resistance (Figure 4A). Although some alleles showed similar trends of resistance for valinomycin and daunorubicin/doxorubicin, others (e.g., S1141T, W1108R, and W1108R-R669C) were qualitatively different in their resistances.

Yeast *MAT* ***a*** *ste6* strains, which lack the **a** factor pheromone transporter, are reported to be more sensitive to actinomycin D than wild-type strains [25]. This prompted us to investigate the interesting possibility that *MAT* ***a*** cells are intrinsically more resistant to actinomycin D than *MATα* cells. Indeed *MATα* cells were dramatically more sensitive to actinomycin D (EC_50_ 15 μg/ml) than *MAT* ***a*** cells (EC_50_ 48 μg/ml). To see if the cytotoxicity profile pattern of P-gp variants is changed with actinomycin D, all variants were tested in a *MAT* ***a*** ste6 strain (JRY8572) for their levels of resistance to actinomycin D (JRY8573–JRY8584) (Figure 4A).

We tested the statistical significance of all comparisons between the reference and each variant for each drug (Table S4). Five variants (S1141T, A893S-R669C, A893S-M89T, L662R, and R669C) exhibited a statistically significant increase in EC_50_ or EC_30_ values for two or more drugs. The A893S and A893S-P1051A variants caused an increase in resistance only for doxorubicin and valinomycin, respectively. The compromising effect of W1108R on R669C was obvious in resistance for all four drugs (Figures 2 and 4A). To see if the relative resistance profile of the P-gp variants to one substrate was predictive of the relative resistance profile to other substrates, we determined the correlation coefficient for all combinatorial pairs of the four relative resistance profiles (Figure 4B). The resistance profiles of three anticancer agents (daunorubicin, doxorubicin, and actinomycin D) were highly correlated to each other, whereas the resistance profile of valinomycin exhibited a relatively low degree of correlation with those of the other three drugs.

## Discussion

To understand the correlation between *ABCB1* polymorphisms and altered cellular pharmacokinetics, we have developed functional assays of P-gp variants in yeast cells. The function of nonsynonymous SNPs was quantitatively measured in isolation from all other variations in the human genome in a yeast-based in vivo assay. The most sensitive measure of drug transport was a colony-counting assay, which provided both qualitative and quantitative measures of drug resistance in yeast expressing reference and variant P-gp. We observed multiple differences caused by the P-gp variants in the level of resistance to the anticancer agents, daunorubicin, doxorubicin, and actinomycin D, and the potassium ionophore valinomycin. The functional consequences of five *ABCB1* polymorphisms were previously unknown: the M89T, L662R, R669C, and S1141T variants were associated with increased resistance to two or more drugs; and the W1108R variant strongly mitigated the impact of R669C on gain of P-gp function (Figures 2 and 4A). Due to its high allele frequency (11% in African Americans), the S1141T SNP in particular deserves further attention to define its clinical significance. As measured by plating efficiency in an acute exposure test, the difference between the reference and most sensitive (W1108R) alleles was approximately 30-fold. In a chronic exposure involving growth in the presence of the drug, like most quantitative comparisons of the activity of single amino acid substituted P-gp mutants in the published data, the differences among the P-gp variant alleles in EC_50_ or EC_30_ values were modest in most cases. The functional variations can be magnified in clinical practice, especially for anticancer agents due to *ABCB1* amplification in cancer patients. In previous studies, the A893 variant, which is the most common SNP, caused either no significant functional impact [20,26,27] or increased P-gp function for digoxin efflux [28]. The data shown here were able to detect an effect of this allele and uncovered unexpected complexity in the response. In the acute assay, A893S cells were significantly more sensitive to both daunorubicin and doxorubicin than cells with the reference allele (Figure 2A and 2B). In contrast, in the chronic assay the A893S allele was indistinguishable from the reference allele with respect to daunorubicin and slightly more resistant to doxorubicin (Figure 2C).

Like variants of facilitated drug influx pumps in the solute-carrier superfamily, P-gp variants that increased function were common. Most random changes in protein sequence are expected to be deleterious or neutral. The significant enhancement of function common to the alleles tested here may reflect a recent adaptation of human populations to local conditions like toxin exposure, leading to selective pressures on medically relevant phenotypes. Interestingly, in Europeans *CYP* genes encoding drug-metabolizing enzymes show strong signals of very recent positive selection [29].

Despite its distinct chemical structure, the resistance profile of actinomycin D showed a high level of correlation with those of the other anticancer agents, daunorubicin and doxorubicin (Figure 4B). Valinomycin, which lowers the mitochondrial membrane potential, inducing apoptosis in some cell lines [30], exhibited a low correlation in resistance relative to other drugs, presumably reflecting differences among P-gp variants in recognition or transport of the drugs. The resistance profiles of the S1141T, W1108R, and W1108R-R669C variants showed the largest variation across substrates. Based on this finding, we speculate that the region containing W1108 and S1141 contributes to the substrate discrimination activity of P-gp. To date, all mutations that alter substrate specificity of P-gp have been located in the transmembrane domains [16]. In contrast, all seven SNPs for which functional consequences were determined in this study are located either in the extracellular region (M89T) or in the cytoplasmic region (the remaining six variants).

We used two widely accepted criteria for predicting the functional effect of uncharacterized SNPs to prioritize for functional characterization (Table 1). Our data on functional consequences revealed that these predictions were sound: four functional SNPs (L662R, R669C, W1108R, and S1141T) scored highly on both criteria, while the two SNPs (A893S and P1051A) that showed no significant functional impact had lower scores on evolutionary conservation and chemical dissimilarity, respectively. One exception was the M89T variant that altered function despite being poorly conserved among mammals.

Most previous functional studies focused on the impact of individual SNPs rather than that of haplotypes. However, in at least some cases, drug response correlates with the patients' haplotypes rather than individual SNPs [31,32]. We tested SNP interactions to see if a compound allele consisting of two SNPs has a unique phenotype different from those of single-SNP alleles. Indeed, it is striking that the strong impact of R669C on P-gp function diminished almost completely when combined with W1108R (Figures 2 and 4A). In contrast, the W1108R variant either alone or with A893S contributed no significant alterations in EC_50_ or EC_30_ values. This result highlighted the importance of testing the impact of all substitutions in a gene together and suggests that compensatory SNPs may exist in nature.

SNPs in the *ABCB1* gene have been implicated in altering drug response or susceptibility to diseases such as Parkinson's disease [33], inflammatory bowel disease [34], and renal epithelial tumors [35]. However, in many such cases, the reported effects of *ABCB1* polymorphisms are conflicting or inconsistent [26,36–38]. This inconsistency may have several causes. First, P-gp expression levels may be modified by nongenetic factors, such as diet and comedications, especially when surgical specimens are studied. Second, previous studies with mammalian cell lines rely on transient expression assays, which swamp the subtle effects of SNPs by variable levels of expression. Third, only a few coding SNPs have been functionally tested, such as A893S and N21D, which our analysis predicted would have a weak functional impact [26]. The use of yeast to evaluate the function of nonsynonymous coding SNPs bypasses these issues and allows the function of single coding SNPs and haplotypes to be assessed directly, independent of all other variations in their original human genome. This “in yeast pharmacogenetics” can function as a robust screening and phenotyping tool to characterize additional SNPs in *ABCB1* and presumably other human multidrug transporter genes.

 During the course of these studies, we observed that *MATα* cells were highly sensitive to actinomycin D, whereas *MAT* ***a*** cells were resistant. This was apparently due to expulsion of the drug by the **a** cell-specific Ste6 transporter. Perhaps chemical exposures in ecological niches or the consequences of treatment with therapeutics might lead to the extreme mating-type biases observed with some fungal pathogens. For example, the mating-type–specific niches occupied by Cryptococcus neoformans may reflect the ability to transport toxins out of the cell in certain environments [39].

## Materials and Methods

### Yeast strains and growth conditions.

S. cerevisiae strains used in this study are listed in Table S1. Standard rich medium (YPD), CSM, and synthetic medium lacking nutritional supplement(s) (CSM–Ura, CSM–His, and CSM–Ura–Trp) were prepared as described [40]. Yeast cells were grown routinely at 30 °C.

### Plasmids.

A P-gp-expressing plasmid, pJR2702 (alias pYKM77; a multicopy-number vector), was kindly provided by Jeremy Thorner (University of California, Berkeley, California, United States) and used for constructing expression plasmids for *ABCB1* bearing different SNPs. A cDNA for the human *ABCB1* coding sequence (GenBank accession number M14758.1) was cloned into a multicopy *URA3*-marked plasmid with the 2 μm origin of replication (YEp352) and expressed from the yeast *STE6* promoter (pJR2702). Substitutions at the SNP position were carried out in pJR2702 by site-directed mutagenesis with primers designed to generate individual haplotypes (Table S5), using the QuikChange site-directed mutagenesis kit from Stratagene (http://www.stratagene.com). We introduced five single SNP alleles and four compound alleles consisting of a two-SNP haplotype into the reference plasmid (pJR2703), creating plasmids pJR2704 to pJR2712 (Table S1). As a negative control, a −1 frameshift mutation at codon 1,200 of the *ABCB1* sequence (1,280 amino acids) was constructed; this cDNA encodes a truncated product of 1,228 amino acids expected to be nonfunctional when expressed (pJR2713). Presence of the desired substitution in the plasmids was verified by DNA sequencing. These eleven constructs, along with another control lacking the entire *ABCB1* sequence (pJR1016), were transformed into a *MAT* ***a*** yeast strain lacking three different ABC transporter genes (*Δpdr5 Δsnq2 Δyor1*, JRY8012), resulting in strains JRY8025 to JRY8036 (Table S1).

### Drug compounds.

Daunorubicin and doxorubicin were kindly provided by Robert Schultz in the Developmental Therapeutics Program of the National Cancer Institute, National Institutes of Health (NIH) (Rockville, Maryland, USA). Valinomycin and actinomycin D were from Sigma (http://www.sigmaaldrich.com). For drug cytotoxicity assays, stock solutions of the drug were prepared at 10 mM in 5% DMSO for daunorubicin and doxorubicin, in 98% ethanol for valinomycin, and in 100% DMSO for actinomycin D.

### Drug cytotoxicity assays.

In the spotting assay, cultures from each strain were grown to midexponential phase, titrated to the same concentration (~10^7^ cells per 1 ml), and serially diluted 5-fold. Aliquots (4 μl) from the dilution series were spotted onto a CSM–Ura plate containing the indicated concentration of the drug. Control plates lacking the drug contained the solvent control at the same concentrations as CSM–Ura plates containing the drug. In the plate assay, cultures from each strain were grown to midexponential phase and titrated to the same concentration (~10^5^ cells per 1 ml). Aliquots (100 μl) were spread onto a CSM–Ura plate containing the indicated concentration of the drug. The same aliquots were further diluted 20-fold (~5,000 cells per 1 ml) and spread onto control plates lacking the drug. After incubation for three days, colony numbers per plate were counted.

Drug resistance was further assayed quantitatively in 96-well microtiter plates (Corning, http://www.corning.com), containing equal volumes (200 μl) of CSM–Ura liquid medium with different concentrations of the drug. Yeast transformants grown to stationary phase in CSM–Ura were diluted to an OD_600_ of 0.1. Equal volumes (200 μl) of these diluted cultures containing increasing concentrations of the drug were added to wells and incubated at 30 °C for 24 h in a Tecan microtiter plate reader. Cell growth was monitored in the absence of the drug in the presence of the same solvent as a negative control. For the experiments with liquid medium, the EC_50_ (median effective concentration) value was defined as the drug concentration that reduced growth of the treated cells to 50% of growth of the control cultures as judged by OD_600_ when the increase in OD_600_ of the control cultures was 0.7 (midexponential phase). To rule out the possibility that variations in copy number affect the observed differences in drug resistance for the vectors bearing each P-gp variant, all measurements were examined in a series of independent transformants for each of the P-gp variants.

### Preparation of membrane extracts.

Membrane fractions of yeast cells with the plasmids bearing *ABCB1* variants (JRY8025–JRY8036) were prepared as described [41].

### Detection and quantification of P-gp variant proteins by immunoblotting.

The mouse monoclonal anti-P-gp antibody C219, kindly provided by Michael Gottesman (National Cancer Institute, NIH, Bethesda, Maryland, United States), was used in immunoblots to quantify the level of P-gp variants in yeast. A rabbit antibody against the Gas1 protein, kindly provided by Randy Schekman (University of California, Berkeley, California, United States), served as a marker of membrane proteins. Human P-gp and yeast Gas1 protein were detected simultaneously on the same blot using infrared-labeled secondary antibodies visualized at two different fluorescence channels, 700 and 800 nm. The blot was developed and quantified by Odyssey Infrared Imaging System (LI-COR Biosciences, http://www.licor.com) following the manufacturer's protocol.

### Construction of GFP-fused P-gp variant strains.

A codon-optimized GFP gene for yeast, yEGFP1 [42], was amplified by PCR with oligonucleotide primers designed to allow in-frame fusion to the 3′ end of *ABCB1* reference and its variants in a yeast expression vector by recombination following transformation into yeast [43]. The presence of yEGFP in the construct was verified by colony PCR and DNA sequencing.

### Visualization of P-gp-GFP fusion proteins.

For fluorescence microscopy, cells were grown in synthetic medium without tryptophan to minimize autofluorescence. Imaging was done at room temperature using an Olympus IX-71 microscope equipped with 100× NA1.4 objectives and Orca-II camera (http://www.olympusamerica.com). ImageJ (http://rsb.info.nih.gov/ij) was used for manipulation of images.

### Statistical analysis.

The probability of a statistically significant difference between the mean values of two datasets was determined by one-way ANOVA with Dunnett's post-test using GraphPad Prism version 4.03 for Windows, GraphPad Software (http://www.graphpad.com).

## Supporting Information

Figure S1 Schematic Secondary Structure of P-Gp and Its Coding Variants The variants were from the dataset of the Pharmacogenetics of Membrane Transporters study [20]. Nonsynonymous SNPs are shown in red and synonymous SNPs are in green. The SNPs prioritized and analyzed in this study are shaded.
(46 KB PPT)

Table S1 Yeast Strains and Plasmids Used in This Study (39 KB DOC)

Table S2 Alignment and Frequency of *ABCB1* Haplotypes Containing Nonsynonymous Variants (92 KB DOC)

Table S3 Survival of Yeast Expressing the P-Gp Variants on a High Concentration of Daunorubicin and Doxorubicin (38 KB DOC)

Table S4 The EC_50_ or EC_30_ Values of the P-Gp Variants for Four Drug Substrates (50 KB DOC)

Table S5 Oligonucleotides Used in Site-Directed Mutagenesis (30 KB DOC)

### Accession Numbers

The Entrez (http://www.ncbi.nlm.nih.gov/Entrez) accession numbers for the genes described in this paper are 5243 for human *ABCB1*, 1576 for human *CYP3A4*, 854324 for yeast *PDR5*, 851574 for yeast *SNQ2*, 853198 for yeast *YOR1*, 853671 for yeast *STE6*, and 855355 for yeast *GAS1*.

The RefSeq (http://www.ncbi.nlm.nih.gov/entrez/query.fcgi?db=OMIM) accession number for human *ABCB1* cDNA carried in plasmid pJR2702 is M14758.1.

The Online Mendelian Inheritance in Man (http://www.ncbi.nlm.nih.gov/entrez/query.fcgi?db=OMIN) accession numbers are 168600 for Parkinson's disease, 266600 for inflammatory bowel disease, and 144700 for renal epithelial tumors.

## Abbreviations

ABC: ATP-binding cassette
*ABCB1,*: ATP-binding cassette transporter B1 gene
CSM: complete synthetic medium
EC: median effective concentration
GFP: green fluorescent protein
*MDR1,*: multidrug resistance 1 gene
P-gp: P-glycoprotein
SNP: single nucleotide polymorphism
