# Supplementary material for: Function-Altering SNPs in the Human Multidrug Transporter Gene ABCB1 Identified Using a Saccharomyces-Based Assay
Source: PLoS Genet. 2007 Mar 9;3(3):e39. doi: 10.1371/journal.pgen.0030039 (PMC1817653; doi:10.1371/journal.pgen.0030039)
Supplement: Supplementary file 1 [file pgen.0030039.st001.doc]

**Table S1.** Yeast strains and plasmids used in this study

| Strain or plasmid | Relevant genotype^a^ | Plasmid feature | Source |
| --- | --- | --- | --- |
| YEp352  pJR2702  JRY6330  JRY6331  JRY8561  JRY8562  JRY8563  JRY8004  JRY8008  JRY8012  JRY8025  JRY8026  JRY8027  JRY8028  JRY8029  JRY8030  JRY8031  JRY8032  JRY8033  JRY8034  JRY8035  JRY8036  JRY8572  JRY8573  JRY8574  JRY8575  JRY8576  JRY8577  JRY8578  JRY8579  JRY8580  JRY8581  JRY8582  JRY8583  JRY8584  JRY8585  JRY8586  JRY8588  JRY8592  JRY8593 | —  —  *MAT****a*** wild-type  *MATα* wild-type  Δ*pdr5*  *Δsnq2*  *Δyor1*  *Δpdr5 Δsnq2*  *Δpdr5 Δyor1*  *Δpdr5 Δsnq2 Δyor1*  *Δpdr5 Δsnq2 Δyor1* YEp352  *Δpdr5 Δsnq2 Δyor1* pJR2703  *Δpdr5 Δsnq2 Δyor1* pJR2704  *Δpdr5 Δsnq2 Δyor1* pJR2705  *Δpdr5 Δsnq2 Δyor1* pJR2706  *Δpdr5 Δsnq2 Δyor1* pJR2707  *Δpdr5 Δsnq2 Δyor1* pJR2708  *Δpdr5 Δsnq2 Δyor1* pJR2709  *Δpdr5 Δsnq2 Δyor1* pJR2710  *Δpdr5 Δsnq2 Δyor1* pJR2711  *Δpdr5 Δsnq2 Δyor1* pJR2712  *Δpdr5 Δsnq2 Δyor1* pJR2713  *Δste6*  *Δste6* YEp352  *Δste6* pJR2703  *Δste6* pJR2704  *Δste6* pJR2705  *Δste6* pJR2706  *Δste6* pJR2707  *Δste6* pJR2708  *Δste6* pJR2709  *Δste6* pJR2710  *Δste6* pJR2711  *Δste6* pJR2712  *Δste6* pJR2713  Δ*pdr5* Δ*snq2* Δ*yor1* pJR2714  Δ*pdr5* Δ*snq2* Δ*yor1* pJR2715  Δ*pdr5* Δ*snq2* Δ*yor1* pJR2717  Δ*pdr5* Δ*snq2* Δ*yor1* pJR2721  Δ*pdr5* Δ*snq2* Δ*yor1* pJR2722 | A multicopy-number vector with a *URA3* marker  *ABCB1* (M14758.1) in YEp352; alias pYKM77  *ABCB1*-free vector  *ABCB1*-reference (haplotype 101) in YEp352  *ABCB1*-A893S (haplotype 102) in YEp352  *ABCB1*-S1141T (haplotype 104) in YEp352  *ABCB1*-A893S, R669C (haplotype 117) in YEp352  *ABCB1*-A893S, M89T (haplotype 118) in YEp352  *ABCB1*-L662R (haplotype 119) in YEp352  *ABCB1*-A893S, P1051A (haplotype 122) in YEp352  *ABCB1*-R669C (haplotype 123) in YEp352  *ABCB1*-W1108R (haplotype 124) in YEp352  *ABCB1*-R669C, W1108R (haplotype 125) in YEp352  A frameshifted *ABCB1* (haplotype 128) in YEp352  yEGFP-fused *ABCB1*-reference (haplotype 101) in YEp352  yEGFP-fused *ABCB1*-A893S (haplotype 102) in YEp352  yEGFP-fused *ABCB1*-A893S, R669C (haplotype 117) in YEp352  yEGFP-fused *ABCB1*-R669C (haplotype 123) in YEp352  yEGFP-fused *ABCB1*-W1108R (haplotype 124) in YEp352 | Lab collection  J. Thorner  Lab collection  Lab collection  Lab collection  Lab collection  Lab collection  This study  This study  This study  This study  This study  This study  This study  This study  This study  This study  This study  This study  This study  This study  This study  This study  This study  This study  This study  This study  This study  This study  This study  This study  This study  This study  This study  This study  This study  This study  This study  This study  This study |

^a^Unless otherwise mentioned, strains were derived from S288C *MAT****a***. JRY6331 has *his3 leu2 ura3 lys2*, JRY8004 had *his3 leu2 ura3 met15 lys2*, and JRY8008 had *his3 leu2 ura3*. All other S288C strains had *his3 leu2 ura3 met15*.
