# Supplementary material for: Function-Altering SNPs in the Human Multidrug Transporter Gene ABCB1 Identified Using a Saccharomyces-Based Assay
Source: PLoS Genet. 2007 Mar 9;3(3):e39. doi: 10.1371/journal.pgen.0030039 (PMC1817653; doi:10.1371/journal.pgen.0030039)
Supplement: Supplementary file 2 [file pgen.0030039.st002.doc]

**Table S2.** Alignment and frequency of *ABCB1* haplotypes containing non-synonymous variants

| Haplotype  number^a^ | Variable amino acid site^b^ | | | | | | | | | | | | | Frequency^c^ | | | | | |
| --- | --- | --- | --- | --- | --- | --- | --- | --- | --- | --- | --- | --- | --- | --- | --- | --- | --- | --- | --- |
|  | 893 | 1141 | 21 | 400 | 261 | 669 | 89 | 662 | 849 | 1051 | 1108 | 1251 | 1256 | CA | AF | AS | ME | PA | Total |
| 101* | A | S | N | S | I | R | M | L | I | P | W | V | T | 97 | 153 | 29 | 10 | 4 | 293 |
| 102* | **S** | S | N | S | I | R | M | L | I | P | W | V | T | 73 | 12 | 26 | 8 | 5 | 124 |
| 103 | **T** | S | N | S | I | R | M | L | I | P | W | V | T | 6 | 1 | 4 | 0 | 5 | 16 |
| 104* | A | **T** | N | S | I | R | M | L | I | P | W | V | T | 0 | 21 | 0 | 1 | 0 | 22 |
| 108 | (**S**) | S | **D** | S | I | R | M | L | I | P | W | V | T | 15 | 4 | 1 | 0 | 0 | 20 |
| 111 | (**S**) | (**T**) | **D** | S | I | R | M | L | I | P | W | V | T | 0 | 1 | 0 | 0 | 0 | 1 |
| 113 | A | S | N | **N** | I | R | M | L | I | P | W | V | T | 4 | 1 | 0 | 0 | 0 | 5 |
| 114 | (**S**) | S | N | **N** | I | R | M | L | I | P | W | V | T | 0 | 1 | 0 | 0 | 0 | 1 |
| 115 | (**T**) | S | N | **N** | I | R | M | L | I | P | W | V | T | 1 | 0 | 0 | 0 | 0 | 1 |
| 116 | A | S | N | S | **V** | R | M | L | I | P | W | V | T | 0 | 3 | 0 | 0 | 0 | 3 |
| 117* | (**S**) | S | N | S | I | **C** | M | L | I | P | W | V | T | 0 | 1 | 0 | 0 | 0 | 1 |
| 118* | **S** | S | N | S | I | R | **T** | L | I | P | W | V | T | 1 | 0 | 0 | 0 | 0 | 1 |
| 119* | A | S | N | S | I | R | M | **R** | I | P | W | V | T | 1 | 0 | 0 | 0 | 0 | 1 |
| 121 | **S** | S | (**D**) | S | I | R | M | L | **M** | P | W | V | T | 1 | 0 | 0 | 0 | 0 | 1 |
| 122* | (**S**) | S | N | S | I | R | M | L | I | **A** | W | V | T | 0 | 1 | 0 | 0 | 0 | 1 |
| 123* | A | S | N | S | I | **C** | M | L | I | P | W | V | T | 0 | 0 | 0 | 0 | 0 | 0 |
| 124* | A | S | N | S | I | R | M | L | I | P | **R** | V | T | 0 | 0 | 0 | 0 | 0 | 0 |
| 125* | A | S | N | S | I | (**C**) | M | L | I | P | **R** | V | T | 0 | 1 | 0 | 0 | 0 | 1 |
| 126 | A | S | N | S | I | R | M | L | I | P | W | **I** | T | 0 | 0 | 0 | 1 | 0 | 1 |
| 127 | (**S**) | S | N | S | I | R | M | L | I | P | W | V | **K** | 1 | 0 | 0 | 0 | 0 | 1 |
|  | S:151  T:17 | T:23 | D:22 | N:7 | V:3 | C:2 | T:1 | R:1 | M:1 | A:1 | R:1 | I:1 | K:1 | 200 | 200 | 60 | 20 | 14 | 494 |

^a^Numbers were assigned based on the minor allele frequency of SNPs. The haplotypes prioritized and analyzed in this study are indicated with (*).

^b^Positions are relative to the transcription start site and based on the cDNA sequence from GenBank accession number M14758.1 with the change V185G. The site of amino acid changes resulting from non-synonymous SNPs is bold-typed. The SNP within parenthesis indicates that it can be on the other chromosome of the same person since the haplotyping is not determined by independently sequencing the two homologous chromosomes. For each SNP, the number of occurrences in the population is indicated at the bottom cell of each column.

^c^Population frequencies are noted. CA, Caucasian-American; AF, African-American; AS, Asian-American; ME, Mexican-American; PA, Pacific Islander; Total, entire sample. Haplotypes 123 and 124 were not found in the population but chosen for our functional analysis.
