# Supplementary material for: Function-Altering SNPs in the Human Multidrug Transporter Gene ABCB1 Identified Using a Saccharomyces-Based Assay
Source: PLoS Genet. 2007 Mar 9;3(3):e39. doi: 10.1371/journal.pgen.0030039 (PMC1817653; doi:10.1371/journal.pgen.0030039)
Supplement: Supplementary file 3 [file pgen.0030039.st003.doc]

**Table S3.** Survival of yeast expressing the P-gp variants on a high concentration of daunorubicin and doxorubicin

| Haplotype number | Relevant SNP(s) | Survived colony numbers for the drug^a^ | |
| --- | --- | --- | --- |
|  |  | Daunorubicin | Doxorubicin |
| 101 | Reference | 502 ± 89 | 559 ± 81 |
| 102 | A893S | **63 ± 5** (< 0.01) | **217 ± 47** (< 0.01) |
| 104 | S1141T | **293 ± 102** (< 0.01) | 395 ± 40 (> 0.05) |
| 117 | A893S-R669C | **198 ± 28** (< 0.01) | 345 ± 60 (> 0.05) |
| 118 | A893S-M89T | **1613 ± 128** (< 0.01) | **1648 ± 226** (< 0.01) |
| 119 | L662R | **720 ± 63** (< 0.01) | **847 ± 127** (< 0.05) |
| 122 | A893S-P1051A | **176 ± 40** (< 0.01) | 330 ± 69 (> 0.05) |
| 123 | R669C | **829 ± 86** (< 0.01) | **1042 ± 169** (< 0.01) |
| 124 | W1108R | **17 ± 10** (< 0.01) | **63 ± 16** (< 0.01) |
| 125 | W1108R-R669C | **137 ± 92** (< 0.01) | **206 ± 64** (< 0.01) |

^a^The values are the average of three independent measurements for the variant ± the standard deviation. The concentrations used for the plate assay were 27.5 μM for daunorubicin and 12.5 μM for doxorubicin. For each drug, differences in colony numbers between reference and each variant were analyzed for their statistical significance by one-way ANOVA with Dunnett’s post test using GraphPad Prism version 4.03. *P* values are presented in parentheses. The values with significant differences (*p* < 0.05) are bold-typed. Values normalized with colony numbers of control plates lacking the drug did not make any significant changes.
