# Supplementary material for: Function-Altering SNPs in the Human Multidrug Transporter Gene ABCB1 Identified Using a Saccharomyces-Based Assay
Source: PLoS Genet. 2007 Mar 9;3(3):e39. doi: 10.1371/journal.pgen.0030039 (PMC1817653; doi:10.1371/journal.pgen.0030039)
Supplement: Supplementary file 4 [file pgen.0030039.st004.doc]

**Table S4.** The EC_50_ or EC_30_ values of the P-gp variants for four drug substrates

| Haplotype number | Relevant SNP(s) | Effective concentration values for the drug (μM)^a^ | | | |
| --- | --- | --- | --- | --- | --- |
|  |  | Daunorubicin | Doxorubicin | Valinomycin | Actinomycin D |
| 101 | Reference | 13.0 ± 0.8 | 8.4 ± 0.6 | 73.5 ± 6.3 | 72.1 ± 3.3 |
| 102 | A893S | 13.3 ± 1.1 (> 0.05) | **10.1 ± 1.0** (< 0.05) | 74.7 ± 5.9 (> 0.05) | 75.8 ± 5.8 (> 0.05) |
| 104 | S1141T | 14.8 ± 1.0 (> 0.05) | **10.8 ± 1.6** (< 0.05) | **118.3 ± 6.5** (< 0.01) | 79.7 ± 2.2 (> 0.05) |
| 117 | A893S-R669C | **16.6 ± 0.9** (< 0.01) | 9.8 ± 0.5 (> 0.05) | **92.3 ± 10.5** (< 0.01) | 81.2 ± 5.5 (> 0.05) |
| 118 | A893S-M89T | **18.4 ± 1.3** (< 0.01) | **12.4 ± 0.8** (< 0.01) | **93.0 ± 4.0** (< 0.01) | 80.4 ± 3.0 (> 0.05) |
| 119 | L662R | 14.9 ± 1.5 (> 0.05) | **11.2 ± 0.6** (< 0.01) | **92.0 ± 8.0** (< 0.01) | 78.0 ± 6.8 (> 0.05) |
| 122 | A893S-P1051A | 13.5 ± 1.3 (> 0.05) | 9.1 ± 1.2 (> 0.05) | **92.3 ± 6.5** (< 0.01) | 75.7 ± 2.5 (> 0.05) |
| 123 | R669C | **17.9 ± 1.8** (< 0.01) | **11.4 ± 1.7** (< 0.01) | **97.7 ± 2.5** (< 0.01) | **84.9 ± 5.6** (< 0.05) |
| 124 | W1108R | 11.9 ± 1.3 (> 0.05) | 8.7 ± 0.6 (> 0.05) | **55.3 ± 11.5** (< 0.01) | 75.2 ± 4.0 (> 0.05) |
| 125 | W1108R-R669C | 13.5 ± 1.2 (> 0.05) | 9.1 ± 1.5 (> 0.05) | **42.0 ± 3.5** (< 0.01) | 73.5 ± 10.9 (> 0.05) |

^a^The values are the average of three or four independent measurements for the variant ± the standard deviation. The values for daunorubicn, doxorubicin, and actinomycin D are EC_50_s; for valinomycin, EC_30_ values are shown. For each drug, differences in effective concentration between reference and each variant were analyzed for their statistical significance by one-way ANOVA with Dunnett’s post test using GraphPad Prism version 4.03. *P* values are presented in parentheses. The EC_50_ or EC_30_ values with significant differences (*p* < 0.05) are bold-typed.
