# Supplementary material for: Function-Altering SNPs in the Human Multidrug Transporter Gene ABCB1 Identified Using a Saccharomyces-Based Assay
Source: PLoS Genet. 2007 Mar 9;3(3):e39. doi: 10.1371/journal.pgen.0030039 (PMC1817653; doi:10.1371/journal.pgen.0030039)
Supplement: Supplementary file 5 [file pgen.0030039.st005.doc]

**Table S5.** Oligonucleotides used in site-directed mutagenesis

| Oligonucleotide  number | Replaced  amino acid^a^ | Sequence 5’ to 3’^b^ | Position^c^ |
| --- | --- | --- | --- |
| oHJ47  oHJ48  oHJ49  oHJ50  oHJ51  oHJ52  oHJ55  oHJ56  oHJ59  oHJ60  oHJ53  oHJ54  oHJ63  oHJ64  oHJ57  oHJ58 | V185G  V185G  A893S  A893S  S1141T  S1141T  R669C  R669C  M89T  M89T  L662R  L662R  P1051A  P1051A  W1108R  W1108R | GATGATGTCTCTAAGATTAATGAAG**GA**ATTGGTGACAAAATTGGAATGTTC  GAACATTCCAATTTTGTCACCAAT**TC**CTTCATTAATCTTAGAGACATCATC  AAGAAAGAACTAGAAGGT**T**CTGGGAAGATCGCTACTG  CAGTAGCGATCTTCCCAG**A**ACCTTCTAGTTCTTTCTT  GCCGGGTGGTG**A**CACAGGAAGAGATCG  CGATCTCTTCCTGTG**T**CACCACCCGGC  CTAATAAGAAAAAGATCAACT**T**GTAGGAGTGTCCGTGGATC  GATCCACGGACACTCCTAC**A**AGTTGATCTTTTTCTTATTAG  GCAGGAAATTTAGAAGATCTG**ACG**TCAAACATCACTAATAGAAGT  ACTTCTATTAGTGATGTTTGA**CGT**CAGATCTTCTAAATTTCCTGC  TTCAAATGATTCAAGATCCAGTC**G**AATAAGAAAAAGATCAACTCGTA  TACGAGTTGATCTTTTTCTTATT**C**GACTGGATCTTGAATCATTTGAA  CGACCGGACATC**GCA**GTGCTTCAGGGA  TCCCTGAAGCAC**TGC**GATGTCCGGTCG  GACTGAATGTTCAG**C**GGCTCCGAGCACAC  GTGTGCTCGGAGCC**G**CTGAACATTCAGTC | 529s  579a  2659s  2695a  3410s  3436a  1984s  2024a  243s  287a  1961s  2007a  3139s  3165a  3308s  3336a |

^a^Positions are relative to the transcription start site and based on the cDNA sequence from GenBank accession number M14758.1.

^b^Modified nucleotides from the original sequence are bold-typed.

^c^Starting position of the primers is followed by ‘s’ in the sense orientation, or ‘a’ in the antisense orientation.
